# Public Disclosure on Social Media of Identifiable Patient Information by Health Professionals: Content Analysis of Twitter Data

**DOI:** 10.2196/19746

**Published:** 2020-09-01

**Authors:** Wasim Ahmed, Reshma Jagsi, Thomas G Gutheil, Matthew S Katz

**Affiliations:** 1 Department of Marketing, Operations and Systems Newcastle University Business School Newcastle University Newcastle upon Tyne United Kingdom; 2 Department of Radiation Oncology Center for Bioethics and Social Science in Medicine University of Michigan Ann Arbor, MI United States; 3 Department of Psychiatry, Beth Israel Deaconess Medical Center, Mass. Mental Health Center Harvard Medical School Harvard University Boston, MA United States; 4 Department of Radiation Medicine Lowell General Hospital Lowell, MA United States

**Keywords:** Social Media, Twitter, Patient Information, Confidentiality, Health Professionals

## Abstract

**Background:**

Respecting patient privacy and confidentiality is critical for doctor-patient relationships and public trust in medical professionals. The frequency of potentially identifiable disclosures online during periods of active engagement is unknown.

**Objective:**

The objective of this study was to quantify potentially identifiable content shared on social media by physicians and other health care providers using the hashtag #ShareAStoryInOneTweet.

**Methods:**

We accessed and searched Twitter’s API using Symplur software for tweets that included the hashtag #ShareAStoryInOneTweet. We identified 1206 tweets by doctors, nurses, and other health professionals out of 43,374 tweets shared in May 2018. Tweet content was evaluated in January 2019 to determine the incidence of instances where names or potentially identifiable information about patients were shared; content analysis of tweets in which information about others had been disclosed was performed. The study also evaluated whether participants raised concerns about privacy breaches and estimated the frequency of deleted tweets. The study used dual, blinded coding for a 10% sample to estimate intercoder reliability using Cohen κ statistic for identifying the potential identifiability of tweet content.

**Results:**

Health care professionals (n=656) disclosing information about others included 486 doctors (74.1%) and 98 nurses (14.9%). Health care professionals sharing stories about patient care disclosed the time frame in 95 tweets (95/754, 12.6%) and included patient names in 15 tweets (15/754, 2.0%). It is estimated that friends or families could likely identify the clinical scenario described in 242 of the 754 tweets (32.1%). Among 348 tweets about potentially living patients, it was estimated that 162 (46.6%) were likely identifiable by patients. Intercoder reliability in rating the potential identifiability demonstrated 86.8% agreement, with a Cohen κ of 0.8 suggesting substantial agreement. We also identified 78 out of 754 tweets (6.5%) that had been deleted on the website but were still viewable in the analytics software data set.

**Conclusions:**

During periods of active sharing online, nurses, physicians, and other health professionals may sometimes share more information than patients or families might expect. More study is needed to determine whether similar events arise frequently and to understand how to best ensure that patients’ rights are adequately respected.

## Introduction

### Background

Physicians, nurses, and other health professionals remain among the most trusted professionals in the United States because of their commitment to the well-being of others; they are a trusted source of health information and guidance [1]. Surveys have demonstrated the high trust in health care professionals of the US public with even higher levels of trust in other countries [1-3]. Still recited by many medical students as they become physicians, the Hippocratic Oath reflects the fundamental importance of patient privacy as a critical element of the doctor-patient relationship and a precondition for the trust of the public. In the United States, the Health Insurance Portability and Accountability Act (HIPAA) requires deidentification of data to avoid sharing protected health information [4]. The US Patient’s Bill of Rights also states that patients have the right to be able to talk privately with medical professionals and that personal information be protected. Thus, both ethical and legal reasons to maintain patient privacy exist.

Fulfilling physicians’ obligations to protect the well-being and privacy of their patients is complicated in the age of the internet. Internet culture is very different from that of the medical profession, creating potential ethical problems with boundaries and privacy that did not exist when physicians interacted exclusively offline. In order to maintain the trust of the public and that of individual patients, physicians increasingly need to understand the limits and risks of disclosure of certain types of information online. Although concerns about unprofessional medical student and resident behavior online have been articulated before [5,6], the ethical risks of public disclosure, when narrative medicine intersects with social media, remain poorly defined [7,8].

Social media usage has become popular among medical professionals. A survey in 2014 by QuantiaMD [9] found that, of 4000 physicians surveyed, 90% noted that they used some form of social media for personal activities, and 65% used social media for professional reasons. In May 2018, thousands of individuals—including many health care professionals—shared health-related stories on Twitter using the hashtag *#ShareAStoryInOneTweet* in response to one physician’s spontaneous tweet of a patient story that included this hashtag. Certain tweets included potentially identifiable information that could be considered a breach of confidence when disclosed without patient consent, risking harm to patients, physician’s careers, and public trust in the profession.

An article in July 2018 [10] highlighted the importance of sharing stories but did not address the potential risks of sharing online. Over time, some viral tweets have been deleted, raising further concerns that the platform allowing easy disclosure might have led to posts that authors subsequently regretted. A notable example of a popular post (altered to avoid identification) was retweeted 13,491 times and liked by 55,994 people before being deleted:

I delivered a baby very underweight, weighing two pounds. They said he did not have a chance. I remained with him for a couple of days. Nine years later, he played his first football game last week.

Hashtags can make online content searchable and discoverable online, regardless of time since publication [11]. The American Medical Association, Massachusetts Medical Society, and other organizations advise physicians to report unprofessional social media use [12,13]. What constitutes unprofessional behavior on social media is not clearly defined. To advance a common understanding and to facilitate subsequent discussion within the profession about what is appropriate, we sought to describe participation of physicians and other health professionals in this event, the reach of their postings, and the occurrence of potentially identifiable disclosures about patients.

### Related Work

Early research on health professionals using Weblogs [14] examined 271 medical blogs, finding that individual patients were described in 114 blogs, and 45 blogs had enough information for patients to identify themselves. Scholars have questioned whether it would ever be ethical for medical professionals to write publicly about patients without their consent [15]. Previous work [16] where young doctors on Facebook were studied has also specifically highlighted privacy issues by finding that some of the private information shared by the doctors (the doctors' own private information shared by the doctors themselves) could bring the profession into disrepute. Previous work [17] has also noted that the use of social networking sites such as Twitter and Facebook by doctors can lead to complaints by patients.

Despite the importance of previous work [16,17], there appears to be a lack of empirical research on the use of popular hashtags for sharing patient stories by medical professionals. Understanding information sharing using hashtags, such as #ShareAStoryInOneTweet, is important because social media is becoming more ingrained in society, and potential privacy violations may exist in this context. Furthermore, as social media use increases, online disclosure of private information via social media is likely to remain an issue for health care systems around the world. However, recent research [18] has also highlighted the positive role medical professionals could play on social media, for instance, by countering medical misinformation.

The results of this study are likely to be of interest to those compiling guidelines for the use of social media by medical professionals.

### Research Questions and Objectives

The overall research aim of this study was to develop a better understanding of the content shared with the hashtag #ShareAStoryInOneTweet.

The objectives of the study were to (1) identify unique tweets sent by doctors and other health care providers using the hashtag, (2) to develop an understanding of the characteristics of the doctors and health care providers using the hashtag, and (3) to categorize tweets into themes and identify the frequency of instances in which patients could be identified by themselves or by their family.

## Methods

### Cohort Definition

Because all information about the published content was publicly accessible, Lowell General Hospital approved this study as institutional review board exempt. To evaluate content in the #ShareAStoryInOneTweet phenomenon, Symplur Signals (Symplur LLC), a proprietary health care–focused database and analytics program collecting data on Twitter using its Enterprise application program interface, was utilized [19]. The first tweet with the hashtag occurred May 4, 2018. From May 4, 2018 through December 31, 2018, 45,040 tweets that included the hashtag #ShareAStoryInOneTweet were identified. The study focused on 43,374 tweets shared in the month of May 2018 (midnight May 1 to midnight June 1, Eastern Standard Time). The analysis was conducted in January 2019. Using the software program, we identified tweets from doctors, patients, and other health care stakeholders (eg, caregivers, pharmaceutical firms, academic, or research organizations), based upon public self-identification in their Twitter profiles (ie, by identifying information provided within their Twitter biography) [20]. There were 4871 tweets identified, of which, 1206 were unique (the remainder represented retweeting of prior postings).

**Figure 1:**
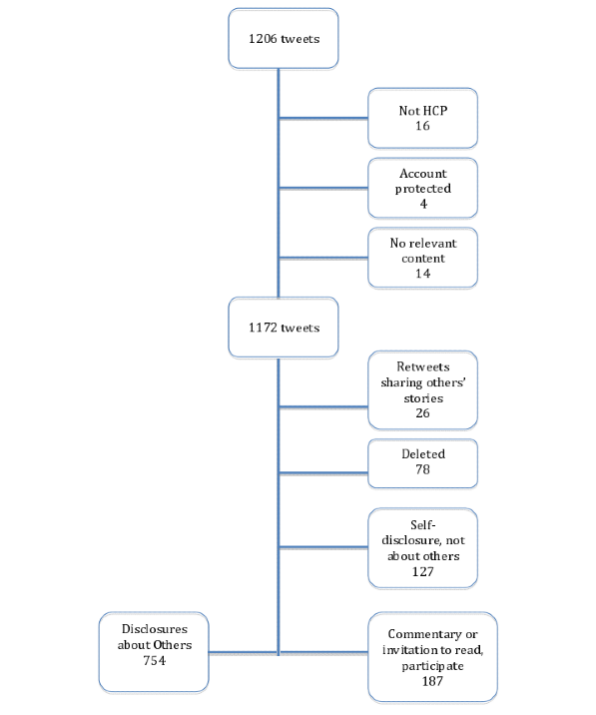
Tweets by doctors and other health care providers during #ShareAStoryInOneTweet in May 2018. HCP: health care providers.

Unique tweets were reviewed by reading text provided within the data set and then evaluating the URL and each account’s public profile on Twitter’s website as of March 2019. Tweets from students misclassified as health care providers (1.3%) (eg, those listing “future doctor” in profile), from blocked accounts (0.3%), or with no relevant content posted with the hashtag (1.2%) were excluded, leaving a total of 1172 tweets shared by physicians, nurses, or other health professionals (Figure 1). The study also excluded 26 retweets in which the authors used the hashtag to share someone else’s disclosure rather than their own, and 127 that included the author’s own illness experiences rather than those of others. The study also excluded 78 tweets (6.5%) with content found in the data set but deleted from Twitter when evaluated on the website.

Characteristics of the health professionals sharing these tweets was examined, using information publicly available in their online profiles, including profession, gender, and country. Physicians were also categorized by specialty as described in their profiles or as unknown if not stated. More detailed content analysis focused upon the tweets in which the health care professional shared the illness or clinical experience of someone other than themselves.

The study also evaluated tweets commenting upon the hashtag-related phenomenon or recommending participation to others. The study analyzed tweets individually rather than as content threads.

### Measures

To assess the magnitude of hashtag use, the study evaluated total number of tweets. We also evaluated the number of total participants, focusing upon physician, nurse, and other health care professionals. We calculated tweets per hour, number of tweets, and use of images both in aggregate and by health care stakeholder categories. In order to capture hourly tweet activity rates, we restricted the time frame to the first two weeks starting May 4th to focus on the viral period. We evaluated the potential reach of the tweets using the software’s definition of *impressions*—follower count at the time of each tweet’s publication online (eg, a doctor posting while having 500 followers was equal to 500 impressions).

For each tweet, we coded several measures: the tweet author’s role in the other person’s clinical care; whether the patient died or was dying; whether the author helped save the other person’s life; inclusion of patient name; inclusion of a clinical image; and inclusion of a specific age. We categorized the time frame of the event described within a tweet as within the past year, 1-2 years ago, 2-5 years ago, >5 years, or unknown.

Whether either a patient or the patient's family or friends would be able to identify the clinical scenario described in each tweet was categorized broadly in response to codebook question “Could patient or family potentially identify the clinical situation?” as *yes* (more likely than not to be identifiable) or *no* (not likely to be identifiable). If it was unclear, the code *indeterminate* was applied and was considered in analyses to be no.

One author (MK) assessed all tweets; a second author (WA) coded a 10% sample independently. Intercoder reliability and percentage agreement were assessed using ReCal [21]. The two authors then reached consensus on discrepancies and used this exercise to identify any areas where the first coder might systematically have erred.

Because the tweets could be discoverable in malpractice or tort suits, we also analyzed whether the author made comments with a negative opinion about the patient or family, or if the author acknowledged that a medical error occurred. We also assessed whether information was shared about vulnerable patients, as defined by the US Department of Health and Human Services [22].

We separately evaluated tweets commenting upon the hashtag to determine whether the authors had a favorable or unfavorable opinion of the viral sharing, or if they invited others to share stories or to participate. We also identified whether these tweets expressed any concern about privacy breaches.

### Statistical Analysis

Overall activity and frequencies for stakeholder participation using Symplur. Frequencies, median, and mean endpoints for content analysis were calculated using Excel (for Mac 2011 version 14.7.2, Microsoft Inc). Cohen κ was used to measure interrater reliability [23].

## Results

### Tweet Volume

**Figure 2:**
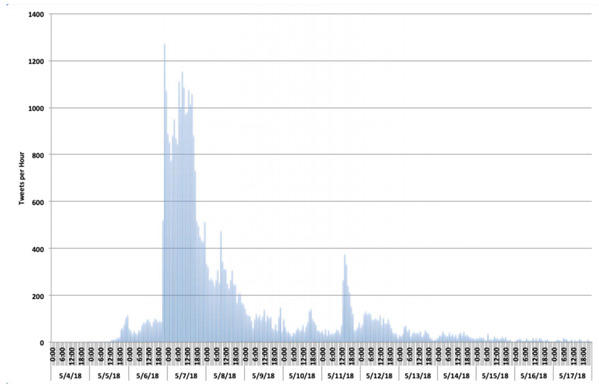
Tweets per hour including the hashtag #ShareAStoryInOneTweet in May 2018.

For May 2018, we identified 31,690 individuals who posted tweets with the hashtag, with a potential of 106.5 million views; 1725 (5.3%) individuals self-identifying as doctors and 861 (2.6%) as other health care providers shared tweets. At its peak, activity showed 1274 tweets per hour (Figure 2).

### Tweets With Disclosures About Others

The characteristics of the health professionals sharing tweets with disclosures about others are presented in Table 1. Of the 656 health professionals, 384 (58.5%) were female; physicians—emergency medicine, family medicine or general practice, and hematology-oncology were the specialties most frequently represented—constituted the largest proportion of the tweeters (486/656, 74.1%), with nurses representing a minority (98/656, 14.9%); and most were in the United States (347/656, 52.9%), followed by Canada (99/656, 15.1%) and the United Kingdom (82/656, 12.5%).

The majority (659/754, 87.4%) involved the sharing of stories about direct patient care, rather than the author’s role as a family caregiver or in another role (Table 2), and 13.6% (95/754) of tweets included a specific time frame. The patient’s age was included in 163 of the 754 tweets (21.6%), and patient name was included in 15 of the 754 (2.0%). Of the 754, 11 tweets (1.5%) shared a clinical image, 152 tweets (20.2%) shared information about people in a vulnerable category. Only, 2 tweets (0.3%) mentioned patient consent to share within the tweet, one explicit and one inferred from past patient agreement to share a specific story. Based upon the number of likes, a minimum of 154,900 accounts viewed these 754 tweets.

Nearly half of the tweets (337/754, 44.7%) described a clinical scenario involving death or dying. Comments disclosing medical errors (6/754, 0.8%) or expressing a negative opinion about the patient or family were rare (4/754, 0.5%). Agreement between coders was 86.8%, and intercoder reliability Cohen κ=0.8 suggested substantial agreement [16]. Disagreements in coding occurred mostly between the categories no unclear, which led to the decision to combine the categories for further analysis. We estimated that almost one-third (242/754, 32.1%) of families or friends would likely find the content in the tweet identifiable. Among patients who were potentially still living, the study estimated that nearly half (162/348, 46.6%) contained likely identifiable information, of which 81 (50%) were likely identifiable by families and friends. The 754 tweets received a median of 2 retweets (range: 0-19; total 959) and 16 likes (range: 0-56; total 735).

**Table 1:** Characteristics of doctors and health care professionals sharing tweets disclosing information about others.

Characteristics			Value (N=656), n (%)
**Gender**			
	Female		384 (58.5)
	Male		266 (40.5)
	Unknown		6 (0.9)
**Profession**			
	**Doctor**		**486 (74.1)**
		Anesthesia	22 (4.5)
		Cardiology	25 (5.1)
		Critical care	12 (2.5)
		Emergency medicine	77 (15.8)
		Family medicine-general practitioner	48 (9.9)
		Gastroenterology	6 (1.2)
		Hematology-oncology	39 (8.0)
		Hospitalist	5 (1.0)
		Infectious disease	5 (1.0)
		Internal medicine	12 (2.5)
		Neurosurgery	5 (1.0)
		Obstetrics and gynecology	13 (2.7)
		Palliative care	18 (3.7)
		Pathology	9 (1.9)
		Pediatrics	28 (5.8)
		Psychiatry	7 (1.4)
		Pulmonary medicine	7 (1.4)
		Radiation oncology/clinical oncology	10 (2.1)
		Radiology	6 (1.2)
		Surgery	19 (3.9)
		Trauma surgery	20 (4.1)
		Unknown	36 (7.4)
		Other	57 (11.7)
	**Nurse**		**98 (14.9)**
		Nurse, not otherwise specified	66 (67.3)
		Critical care	16 (16.3)
		Emergency medicine	8 (8.2)
		Other	8 (8.2)
	Nurse practitioner		12 (1.8)
	Paramedic		18 (2.7)
	Pharmacist		7 (1.1)
	Physical therapy		10 (1.5)
	Psychologist		5 (0.8)
	Social worker		7 (1.1)
	Speech therapy		5 (0.8)
	Other		8 (1.2)
**Country**			
	Australia		11 (1.7)
	Canada		99 (15.1)
	India		7 (1.1)
	Ireland		16 (2.4)
	Saudi Arabia		7 (1.1)
	South Africa		4 (0.6)
	Spain		4 (0.6)
	United Kingdom		82 (12.5)
	United States		347 (52.9)
	Unknown		55 (8.3)
	Other		24 (3.7)

**Table 2:** Content characteristics tweets with disclosures about others.

Content characteristic			Value (N=754), n (%)	
**Author role**				
	Health care professional			669 (88.7)
	Patient			0 (0.0)
	Caregiver			42 (5.6)
	Other			43 (5.7)
**Time frame described**				
	Within past year			5 (0.7)
	1-2 years			6 (0.8)
	2-5 years			5 (0.7)
	> 5 years			79 (10.5)
	Unknown/not described			659 (87.4)
**Content**				
	Author involved in patient care			635 (84.2)
	Dying patient or patient death			337 (44.7)
	Saving a patient’s life			131 (17.4)
	Include a clinical image			11 (1.5)
	Include patient name			15 (2.0)
	Provide specific patient age			163 (21.6)
	Express negative opinion of patient or family			4 (0.5)
	Mention medical error			6 (0.8)
**Estimated likely**				
	Can family or friends identify situation described?			242 (32.1)
	**Can patient identify situation described?**			
		All tweets	183 (24.3)	
		Potentially living patients (n=348)	162 (46.6)	
Vulnerable population			152 (20.2)	

### Tweets Relating to the Hashtag

Of 187 tweets actively part of the conversations without disclosures, 173 made some commentary: 6 tweets (3.2%) raised concerns about privacy or identifiable information in the tweets with disclosures, 1 (0.6%) tweet involved another critical comment, and 167 (96.5%) tweets were neutral or favorable. Of 187 tweets, 42 tweets (22.5%) invited others to read the hashtag’s stream or contribute to it.

## Discussion

### Principal Findings

This retrospective study describes a physician-initiated event sharing health-related stories and information on Twitter by quantifying the global participation of health care professionals and the type of content shared. The tweeted stories became widely shared, attracting media attention and disseminating the information widely. Almost none (either explicitly or appear to) confirm consent to share information publicly on the popular social network. Nurses, physicians, and other health professionals commenting using the hashtag were more likely to express support for the event and encourage others to participate than they were to raise concerns about patient privacy breaches. However, recent research suggests that 12% of patients may have less trust in physicians describing patient stories on social media, even if shared respectfully [24].

The study showed a relatively high incidence of sharing stories including details that might make them potentially identifiable to patients themselves or to families and friends in a setting that involved a large number of health care professionals. This finding highlights a lack of awareness about the privacy issues intrinsically connected to interactions on social media. Early in the use of social media, most US state medical boards received at least one report of an episode of online professionalism violations for disciplinary action, including violations of patient confidentiality [25]. Although surveys of medical students and physicians suggest the incidence of unprofessional behavior among medical students is infrequent [26,27], this study indicates that in some circumstances health care professionals may share more information publicly than the public might expect. Privacy breaches risk potential negative effects on physician-patient relationships, professional disciplinary actions or torts, and eroding public trust.

The findings of this study differ from those of prior studies [16,17] of online medical professionalism at least partly because we analyzed, in detail, one specific event focused upon health-related disclosures. There is no indication this episode was planned, and the incidence of similar episodes is unknown. However, it was not an isolated event; for example, another prominent example involved physicians opposing gun violence, who used the hashtag #ThisIsOurLane on Twitter in November 2018 [28]. Physicians focused on policy issues, but some may have failed to recognize privacy concerns, publishing tweets with photographs similar in nature to prior social media content in other cases, resulted in professional termination [29,30]. Social media studies publishing tweets often permit reverse identification of the authors [31], and a survey suggests that most participants are somewhat or very uncomfortable with their tweets being quoted in a published research paper [32]. It was found that 6.5% of tweets archived in the software’s archived data set were in fact deleted by health care professionals, indicating that some did not want their tweets to remain publicly visible. Even if deleted online, tweets may retain permanence and discoverability, when published in journals.

Most research evaluating online disclosures focus on the privacy paradox, in which people value their privacy but still share their own information. Surveys indicate people may value short-term social rewards of self-disclosure online more than long-term privacy concerns [33], and high social capital of social network users is associated with increased self-disclosures over time [34]. For people disclosing information about others, the research is more limited, but opinion leadership and female gender have been linked to less concern about others’ privacy [35], consistent with the findings of this study. Health care professionals may be prone to these same tendencies, despite their training and education to maintain privacy. Generational differences in concerns about privacy online may also play a role [36], but assessment of this possibility was not within the scope of this study.

Based upon the temporal pattern of sharing, this hashtag-related event may be less similar to narrative medicine and writing and more similar to a brief episode of social contagion, in which viral sharing of content or emotions online may occur and involve more than simple, conscious risk–reward tradeoffs [37,38]. Unlike traditional peer-reviewed publication of a medical story in narrative medicine, tweeting occurs quickly and does not permit editing. The observation that 6.5% chose later to delete their contributions may suggest that some health care professionals who participated in the experience may have later viewed their behavior as a temporary lapse in judgment.

Another contributing factor may be a knowledge gap for physicians and other health care professionals on how to behave online. While many recognize the importance of online professionalism, curricula for use in formal medical education are only beginning to emerge and remain uncommon [39-41]. Of note, the ethical obligation to maintain confidentiality does not end with a patient’s death [42]. The digital medium does not avoid the potential that disclosures about patients risk breaching confidentiality, undermining trust within that therapeutic relationship as well as public trust in the medical profession. The findings of this study suggest a potential need for evidence-based training in ethical digital communications skills for undergraduate, graduate, and continuing medical education. Professional societies could create resources that allow social media authors to document having obtained consent, so that disclosing identifiable patient information without consent does not inadvertently become normalized.

### Limitations

This study had several limitations. First, this study examined a very specific event that may occur during very active periods of online engagement but likely overestimated the general incidence of online behaviors that could, in some cases, constitute violations of medical professionalism. Future research could analyze a broader collection of social media posts by medical professionals. Second, the study could not assess the number of people actually seeing these tweets; only the number of likes was measured, and the potential reach was estimated. Third, the study only analyzed tweets from accounts that the software identified as health care professionals. The evaluation of all tweets in the cohort confirms the software rarely misclassified nonprofessionals into this group, but the study did not evaluate any other participants in the event to determine if the study could identify more participating health care professionals not categorized as doctors or nurses by the software, which could decrease or increase the incidence of potential privacy breaches. Fourth, by analyzing only tweets with the hashtag, the study potentially underestimated the frequency of others expressing concern about patient privacy. Fifth, given the brevity inherent to the medium of Twitter, it is possible that some authors did indeed have formal documentation of patients’ consent to share their stories but that there was insufficient room to include due to character limits in each post. Finally, the assessment of identifiability in this study may differ from those in other studies, and we cannot exclude the possibility that some physicians and nurses tweeting what seemed to be identifiable stories consciously changed important details to deidentify. It was beyond the scope of this study to confirm whether any harm occurred.

Despite these limitations, the findings of this study clearly show that internet-based sharing raises potential pitfalls for medical professionalism. The internet provides nurses, physicians, and other professionals the opportunity to help or harm others on a global scale. Although internet culture may favor maximizing transparency, it can also pose the risk of directly contradicting health professionals’ fiduciary duty: first, do no harm, including harm that may be inflicted by what we say.

### Conclusion

The study identified a high incidence of potential privacy breaches online. More research is essential to confirm the findings of this study and determine how to ensure physicians, nurses, and other professionals adapt their behavior to maintain medical professionalism in the digital age. Our results suggest that some who were using the hashtag may not have appreciated that the information being shared might breach patients’ privacy We recommend greater specification of professional ethical standards in this context along with evidence-based training in ethical digital communications skills for the undergraduate, graduate, and continuing medical education.
